# Domain Swap Approach Reveals the Critical Roles of Different Domains of SYMRK in Root Nodule Symbiosis in *Lotus japonicus*

**DOI:** 10.3389/fpls.2018.00697

**Published:** 2018-06-05

**Authors:** Hao Li, Mengxiao Chen, Liujian Duan, Tingting Zhang, Yangrong Cao, Zhongming Zhang

**Affiliations:** State Key Laboratory of Agricultural Microbiology, College of Life Sciences and Technology, Huazhong Agricultural University, Wuhan, China

**Keywords:** *Lotus japonicus*, root nodule symbiosis, symbiosis receptor kinase, symbiotic signaling, domain swap, nitrogenase activity

## Abstract

Symbiosis receptor kinase (SYMRK) is a cell membrane-localized protein kinase containing extracellular malectin-like domain (MLD) and leucine-rich repeat (LRR) domains, which is critically required for both root nodule symbiosis (RNS) and arbuscular mycorrhizal symbiosis (AMS). SYMRK is widely distributed in the genomes of different plant species; however, the contribution of different domains of SYMRK and its homologs from other plant species to RNS is largely unclear. In this study, SYMRK and its homologs from three typical plant species including *Medicago truncatula* (for both RNS and AMS), *Oryza sativa* (for AMS but not RNS), and *Arabidopsis thaliana* (for neither RNS or AMS) were investigated using domain swap approach in response to rhizobia in *Lotus japonicus*. Full-length SYMRK from rice and *Medicago* but not from *Arabidopsis* could complement *Lotus symrk-409* mutant plants to contribute RNS. The chimeric protein with the extracellular domain (ED) of LjSYMRK and cytoplasmic domains (CD) of SYMRK from both *Medicago* and rice but not *Arabidopsis* could contribute to RNS in *Lotus*, suggesting that the CD of SYMRK is required for symbiotic signaling. The chimeric receptors containing the CD of LjSYMRK (SYMRK^CD^) and the EDs of MtDMI2 (MtDMI2^ED^), OsSYMRK (OsSYMRK^ED^), AtSYMRK (AtSYMRK^ED^), NFR1 (NFR1^ED^), and NFR5 (NFR5^ED^) could complement *Lotus symrk-409* mutant plants to develop nodules. However, MtDMI2 could partially complement *Lotus symrk-409* mutants to form both effective nodules and ineffective bumps, which is similar to the complementation results from MtDMI2^ED^-LjSYMRK^CD^ and LjSYMRK^GDLC^ in *Lotus symrk-409* mutants, suggesting that ED of SYMRK has a very fine-tune regulation for RNS in *Lotus*. The deletion of either MLD or LRR on SYMRK^GDLC^ (a mutant version of SYMRK with GDPC motif replaced by GDLC) could contribute to RNS when overexpressed in *Lotus symrk-409* mutants, suggesting that MLD and LRR domains might work together to be involved in symbiotic signaling and the LRR domain might play a negative role in LjSYMRK^GDLC^-mediated RNS. By mutagenizing the conserved amino acids on LRR domain, five serine residues were found to be required for the function of LjSYMRK^GDLC^ in RNS. These finding precisely refine the molecular mechanisms of SYMRK function in symbiotic signaling in *L. japonicus*.

## Introduction

In barren soils, leguminous plants form symbiosis with rhizobia leading to the development of a new organ called a nodule, where rhizobia reside and subsequently reduce nitrogen into ammonium for the plant host in exchange for nutrients. In comparison to the root nodule symbiosis (RNS), which is mostly restricted in legumes, the arbuscular mycorrhizal symbiosis (AMS) is widespread in most landed plants, with a few exceptions (for example cruciferous plants). It is estimated that AMS originated about 400 million years ago, while RNS originated about 60 million years ago (Remy et al., 1994; Young et al., 2011), consistent with the generally accepted theory that RNS evolved from AMS and both of them evolved from plant–pathogens interaction (Parniske, 2008; Markmann and Parniske, 2009).

For almost all the symbiosis between legumes and rhizobia, the initiation of compatible interaction begins with a molecular dialog between two partners (Oldroyd, 2013). The flavonoids secreted by plant hosts induce the biosynthesis and secretion of lipo-chitooligosaccharide called Nod factor (NF) by rhizobia (Fisher and Long, 1992). In the root hairs, two LysM receptor-like kinases, for example, NFR1/NFR5 in *Lotus*, or LYK3/NFP in *Medicago*, could recognize the NF to initiate the symbiotic signaling leading to the formation of root nodules for rhizobial colonization (Limpens et al., 2003; Madsen et al., 2003; Radutoiu et al., 2003; Arrighi et al., 2006; Broghammer et al., 2012; Moling et al., 2014). Recently, another LysM-RLK (exopolysaccharide receptor 3, EPR3) was shown to recognize rhizobia EPS, indicating that plants could recognize at least two symbiotic signals to mediate symbiosis (Kawaharada et al., 2015, 2017). A leucine-rich repeat (LRR) receptor-like kinase, Lotus SYMRK or Medicago Does Not make Infections (DMI2), plays a key role in symbiotic signaling acting as a downstream component of Nod Factor Receptors (Ané et al., 2002; Stracke et al., 2002; Miwa et al., 2006). All these receptor-like kinases are required for calcium spiking and expression of symbiosis-relate genes *via* a common symbiosis pathway (CSP) (Oldroyd and Downie, 2008; Kouchi et al., 2010). Similar to RNS, LjNFR1/MtLYK3 also participates in AMS with a possible function to recognize Myc Factor produced by arbuscular mycorrhizal fungi (Zhang et al., 2015). The Myc and Nod factors initiate a common signaling pathway (CSP) with overlapped signaling components identified (Oldroyd, 2013; Genre and Russo, 2016). Symbiosis receptor kinase (SYMRK) is widespread in most plant species such as leguminous and cereal plants. Furthermore, in the *Arabidopsis* who could not have the capability of either RNS or AMS has two SYMRK homologs, i.e., AT1G67720.1 and AT2G37050.1 (Shiu and Bleecker, 2001).

In *Lotus*, knock-out of SYMRK completely abolished the ability to form ITs or produce nodules with rhizobia. However, in the *symrk-14* mutant plants that harbor a point mutation at GDPC motif linking malectin and LRR domains, epidermal responses including infection thread formation were significantly reduced, while the formation of nodule primordia and cortical infection were slightly changed (Kosuta et al., 2011), suggesting that epidermal response and cortical program might be differentially regulated during symbiotic interaction with rhizobia. Since GDPC motif was shown to be required for the cleavage of malectin-like domain (MLD) from SYMRK protein (Antolín-Llovera et al., 2014), malectin and LRR might have a fine-tune regulation mechanism in mediating symbiotic response. The truncated version SYMRK^ΔMLD^ (lacking the MLD) was shown to be able to interact with NFR5 and transfer the symbiotic signal to downstream targets (Antolín-Llovera et al., 2014). In *Lotus*, overexpression of the full-length SYMRK could produce spontaneous nodules in the absence of rhizobia (Ried et al., 2014). Similarly, overexpression of the kinase domain of *Arachis hypogaea* SYMRK (AhSYMRK^KD^) could also induce spontaneous nodule formation in the absence of rhizobia (Saha et al., 2014), suggesting that the kinase domain of SYMRK might have a dominant positive role in nodule organogenesis. However, the autophosphorylation of SYMRK seems not to be essential to mediate symbiotic signaling, since mutation at an autophosphorylation site (Tyr-670) in AhSYMRK could partially complement *symrk* mutants by producing infection threads and uninfected nodule primordia (Samaddar et al., 2013; Paul et al., 2014; Saha et al., 2016). In addition, expression of *OsSYMRK* under the control of the *SYMRK* promoter could only complement *symrk* mutants to produce AMS but not RNS in *Lotus* (Markmann et al., 2008). All these results not only indicated the critical role of SYMRK protein in RNS, but also suggested that SYMRK uncouples the rhizobia infection and nodule formation. However, how the extracellular domains (EDs) of SYMRK is involved in RNS is largely unknown.

In this study, we demonstrate that MtDMI2 (SYMRK homolog protein in *Medicago*) and OsSYMRK but not AtSYMRK could complement *Lotus SYMRK*^−/−^ plants. In addition, multiple chimeric SYMRK proteins were developed to analyze the function of different domains of SYMRK in RNS. The data refine the molecular mechanisms of each domain of SYMRK in RNS in *Lotus*.

## Results

### Homology analysis of SYMRK in legumes and non-legumes

**Figure 1:**
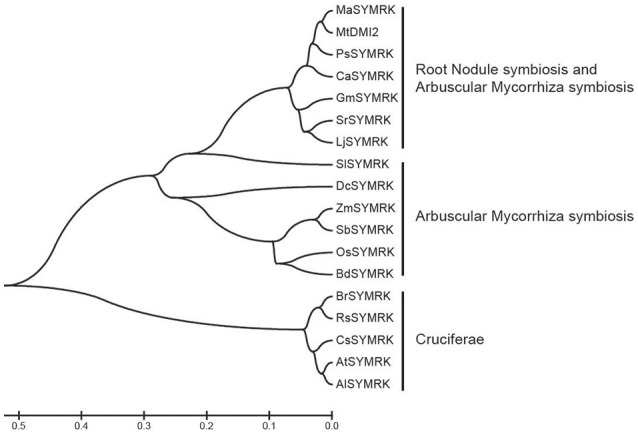
Phylogenetic tree of SYMRK proteins in different species. The Phylogenetic tree was constructed using MEGA 5.1 software with 1,000 bootstrap replications. Abbreviation of each species names were used to represent the SYMRK protein cloned from this species. Ma, *Melilotus albus*; Mt, *M. truncatula*; Ps, *Pisum sativum*; Ca, *Cicer arietinum*; Gm, *Glycine max*; Sr, *Sesbania rostrata*; Lj, *L. japonicus*; Sl, *Solanum lycopersicum*; Dc, *Dendrobium catenatum*; Zm, *Zea mays*; Sb, *Sorghum bicolor*; Os, *Oryza sativa*; Bd, *Brachypodium distachyon*; Br, *Brassica rapa*; Rs, *Raphanus sativus*; Cs, *Camelina sativa*; At, *Arabidopsis thaliana*; Al, *Arabidopsis lyrata*.

SYMRK is critically required for both root nodule symbiosis (RNS) and AMS. To study its function involved in RNS, SYMRK homologs were identified based on sequence similarity with *Lotus* SYMRK using BLASTp as a search engine at the National Center for Biotechnology Information database (NCBI), and 18 SYMRK homologs were identified. A phylogenetic tree based on various SYMRK proteins was made using the MEGA 5.1 software (Figure 1). In the phylogenetic tree, SYMRK proteins and homologs fall into three different clades. Clade I contains SYMRK proteins from plants that could have both RNS and AMS; clade II contains SYMKR from plants having AMS but not RNS, while proteins from Cruciferae plants that could not possess either AMS or RNS fallen into clade III.

### Both OsSYMRK and MtDMI2 but not AtSYMRK could complement *symrk-409* mutant in *Lotus*

To study the function of SYMRK involved in RNS in *Lotus*, an *LORE1*-insertion *symrk* mutant were identified from the Centre for Carbohydrate Recognition and Signaling (CARB, http://users-mb.au.dk/pmgrp/) (Fukai et al., 2012; Urbanski et al., 2012; Małolepszy et al., 2016). The line 30010361 (*symrk-409*) contains an *LORE1*-insertion between exon 4 and intron 4, leading to the early termination of translation of SYMRK (Figures S1A–C). In response to rhizobia treatment, infection threads and nodules were not detected in the *symrk-409* mutant plants when compared with *Lotus* B-129 Gifu 7- and 21-day post-inoculated (DPI) with rhizobia (Figures S1D–G), indicating that the *symrk-409* is another knock-out mutant that could be used for future study.

To analyze the symbiotic function of various SYMRK proteins, one SYMRK protein from each clade-based phylogenetic tree, respectively, was chosen for study, i.e., MtDMI2 from *Medicago* that could have both RNS and AMS, OsSYMRK from rice that could only have AMS, and AtSYMRK from *Arabidopsis* that could have neither RNS or AMS.

**Figure 2:**
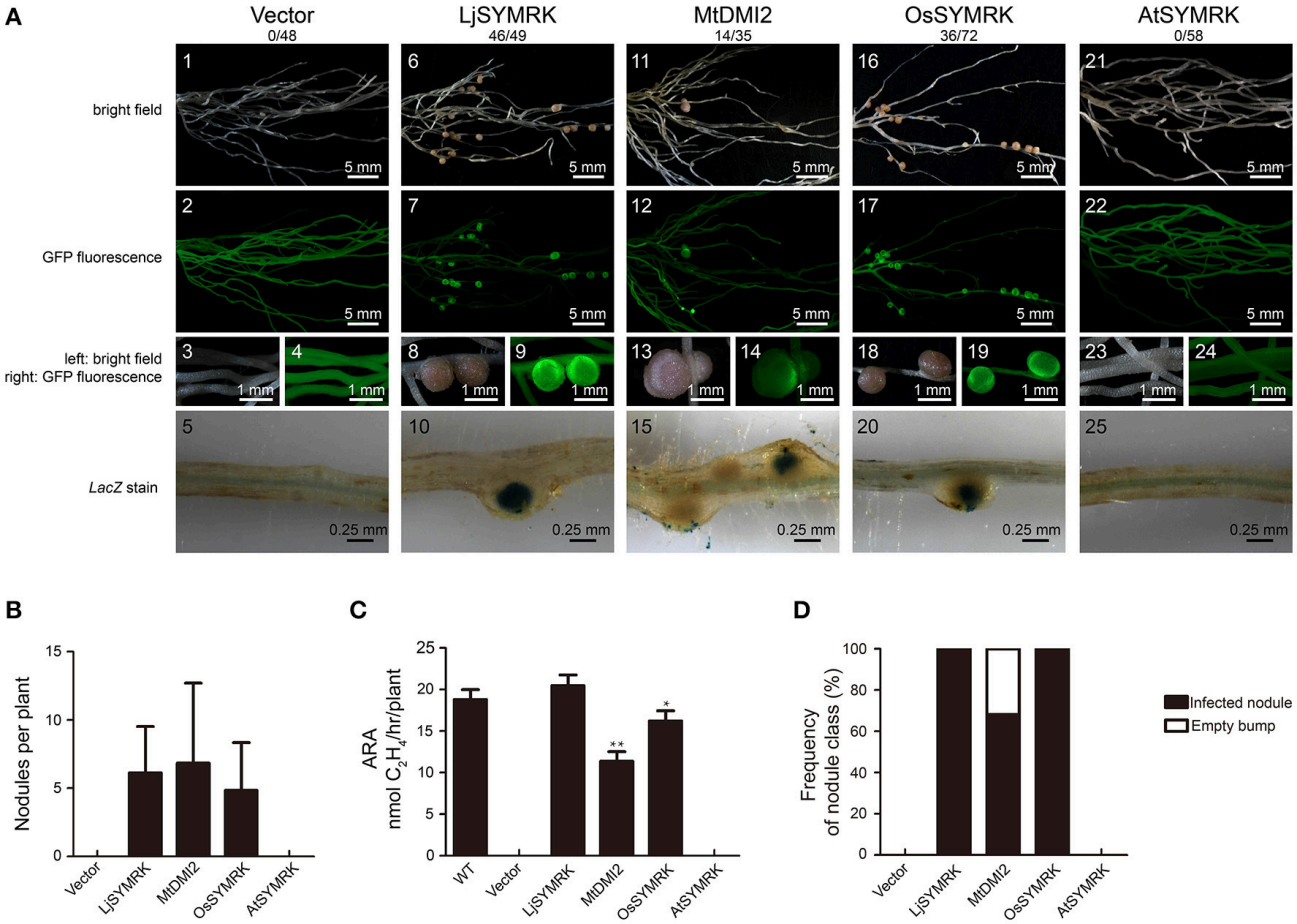
Complementation of *Lotus symrk-409* mutants with LjSYMRK, MtDMI2, OsSYMRK, and AtSYMRK. **(A)** Representative micrographs of different transgenic nodules. Positive transgenic roots identified using GFP signals were inoculated with *M. loti* strain NZP2235 containing a *LacZ* report gene. Nodules and bumps were stained with X-gal. Images showed nodules produced on *symrk-409* roots transgenically expressed with empty control **(A1–5)**, *LjSYMRK* **(A6–10)**, *MtDMI2* **(A11–15)**, *OsSYMRK* **(A16–20)**, and *AtSYMRK* **(A21–25)**. Digital numbers on top of **(A)** indicate the number of nodulated plants out of the positive transgens. **(B)** Numbers of nodules and bumps generated on different transgenic roots 21 DPI with rhizobia. **(C)** Nitrogenase activity of root nodules 21 DPI with rhizobia determined using acetylene reduction method. ^*^P < 0.05, ^**^P < 0.01 indicate significant differences (*t*-test). **(D)** Frequency of nodules and bumps produced on the transgenic roots.

To test the function of each SYMRK as indicated above, SYMRK proteins including LjSYMRK, MtDMI2, OsSYMRK, and AtSYMRK and control vector were transgenically expressed in the *symrk-409* mutant plants under the control of the *Ljubiquitin* promoter using hairy root transformation. The *Ljubiquitin* promoter could induce a strong expression of multiple genes in previous publications (Maekawa et al., 2009; Yoro et al., 2014; Wang et al., 2016). Compared with no nodules observed in the control transformation (Figures 2A1–5), overexpression of LjSYMRK or OsSYMRK or MtDMI2 but not AtSYMRK-induced nodule formation 21 DPI with rhizobia in *symrk-409* mutant plants (Figure 2A). There was no significant difference in the number of nodules per plant root transformed with either LjSYMRK or MtDMI2 or OsSYMRK 21 DPI with rhizobia (Figure 2B). In comparison to wild type and LjSYMRK-transgenic *Lotus* nodules, decreased nitrogenase activities from transgenic nodules with expression of either MtDMI2 or OsSYMRK were measured. While no nitrogenase activity was detected in roots transformed with either vector control or AtSYMRK (Figure 2C). In consistence with the low levels of nitrogenase activity from MtDMI2-transgenic nodules, about 30% ineffective bumps with no rhizobia infection were observed (Figure 2D). However, no ineffective bumps were observed in OsSYMRK-transgenic Lotus *symrk-409* mutant roots. These data indicate that both MtDMI2 and OsSYMRK but not AtSYMRK could complement *symrk-409* mutant to form nodules.

Since both *Lotus* and *Medicago* belong to leguminous plants, we examined whether LjSYMRK could complement *MtDMI2*-knock-out mutant plants *dmi2-1* (or named TR25). Both LjSYMRK and MtDMI2 and control vector were introduced into *dmi2-1* using hairy root transformation. In comparison with no infection threads and nodules observed on the *dmi2-1* mutant transformed with empty vector (Figures S2A1–5,B), a significant number of nodules and infected nodule primordium was detected on roots transformed with either MtDMI2 or LjSYMRK (Figures S2A6–15,2B), which is consistent with nitrogenase activity measured in these two transgenic nodules (Figure S2C). However, about 20% nodule cases formed on *dmi2-1* mutant plants expressing *LjSYMRK* are ineffective since no rhizobia were detected using lacZ staining (Figure S2D), suggesting that MtDMI2 and LjSYMRK have overlapping function to complement each other but with exceptional roles in mediating symbiosis in their own hosts.

### The CD of SYMRK is essential but the ED is important in RNS in *Lotus*

**Figure 3:**
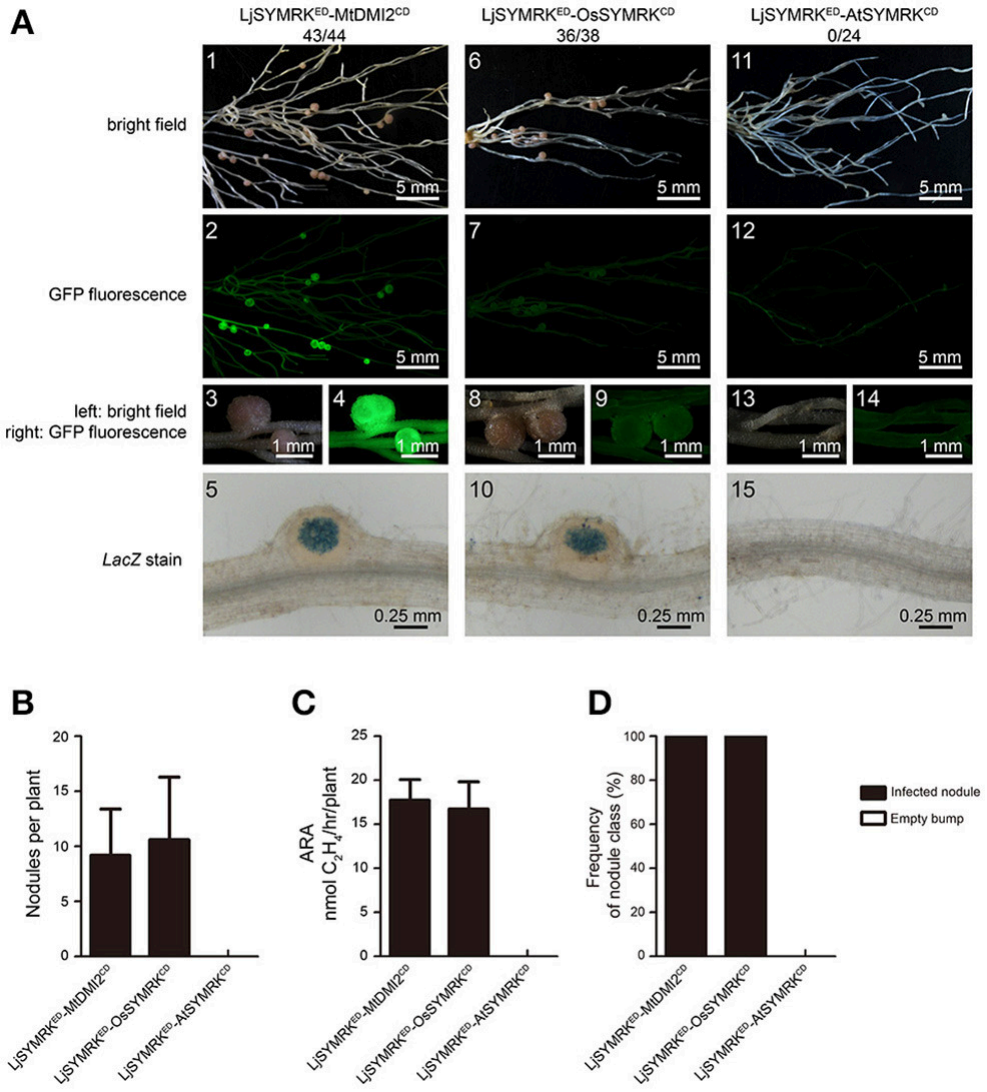
Complementation of *Lotus symrk-409* mutants with LjSYMRK^ED^-MtDMI2^CD^, LjSYMRK^ED^-OsSYMRK^CD^, and LjSYMRK^ED^-AtSYMRK^CD^. **(A)** Representative micrographs of different transgenic nodules. Positive transgenic roots identified using GFP signals were inoculated with *M. loti* strain NZP2235 containing a *LacZ* report gene. Nodules and bumps were stained with X-gal. Images showed nodules produced on *symrk-409* roots transgenically expressed LjSYMRK^ED^-MtDMI2^CD^ **(A1–5)**, LjSYMRK^ED^-OsSYMRK^CD^ **(A6–10)**, and LjSYMRK^ED^-AtSYMRK^CD^ **(A11–15)**. Digital numbers on top of panel A indicate the number of nodulated plants out of total positive trasngens. **(B)** Numbers of nodules produced per transgenic roots 21 DPI with rhizobia. **(C)** Nitrogenase activities of root nodules 21 DPI with rhizobia determined using acetylene reduction method. **(D)** Frequency of nodules and bumps generated on the transgenic roots.

To study the function of each domain of SYMRK from different plant species, a variety of constructs was made using domain swaps and functionally tested in *symrk-409* mutant plants using hairy root transformation (Figure S3). To study the function of the cytoplasmic domain (CD) of SYMRK, the CD of LjSYMRK replaced with CDs of MtDMI2, OsSYMRK, and AtSYMRK (Figure S3). In *symrk-409*, roots transformed with LjSYMRK^ED^-MtDMI2^CD^ and LjSYMRK^ED^-OsSYMRK^CD^ formed normal infected nodule primordia and effective nodules (Figure 3A1–10,B–D) with similar nitrogenase activities measured (Figure 3C). However, LjSYMRK^ED^-AtSYMRK^CD^ could not complement *symrk-409* to form infection threads and nodules (Figures 3A11–15). These data suggest that the CD of SYMRK plays an essential role in RNS.

**Figure 4:**
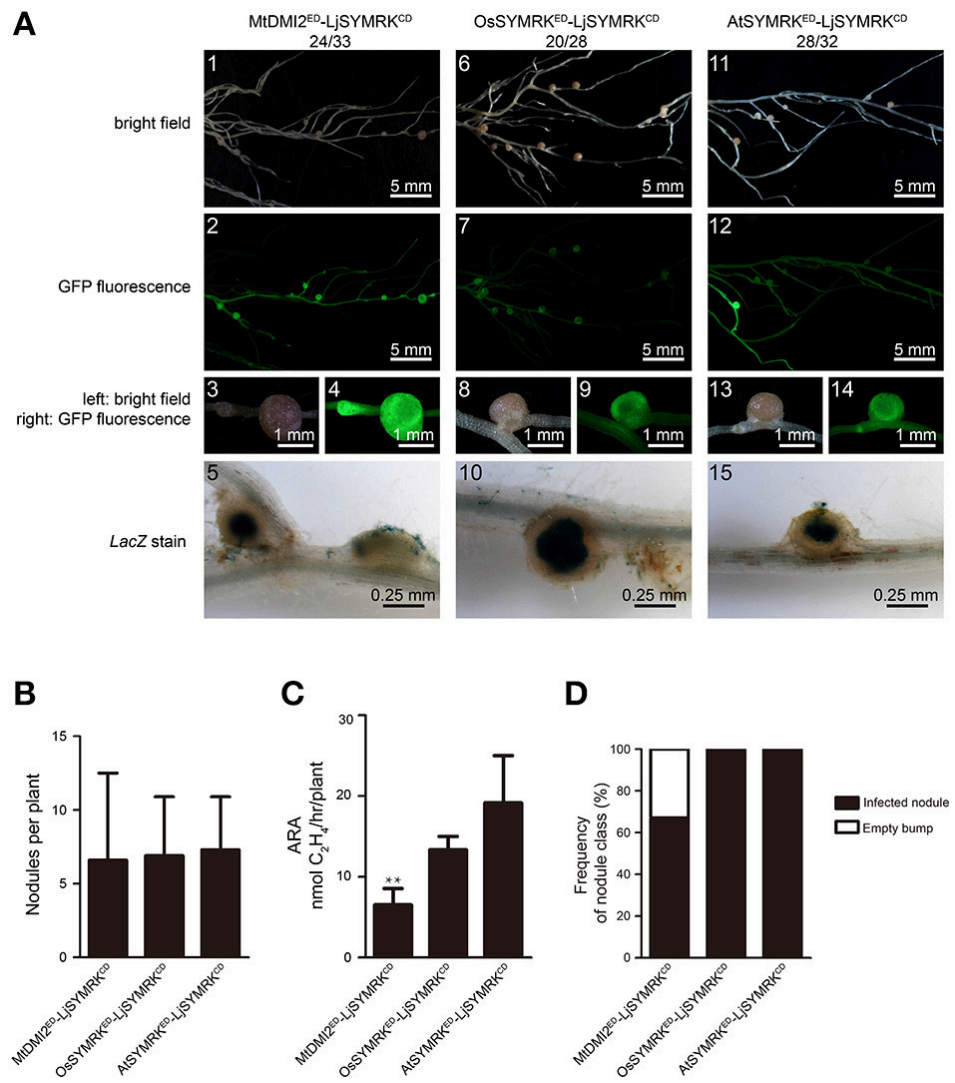
Complementation of *Lotus symrk-409* mutants with MtDMI2^ED^-LjSYMRK^CD^, OsSYMRK^ED^-LjSYMRK^CD^, and AtSYMRK^ED^-LjSYMRK^CD^. **(A)** Representative micrographs of different transgenic nodules. Positive transgenic roots identified using GFP signals were inoculated with *M. loti* strain NZP2235 containing a *LacZ* report gene. Nodules and bumps were stained with X-gal. Images showed nodules produced on *symrk-409* roots transgenically expressed MtDMI2^ED^-LjSYMRK^CD^ **(A1–5)**, OsSYMRK^ED^-LjSYMRK^CD^ **(A6–10)**, and AtSYMR^ED^-LjSYMRK^CD^ **(A11–15)**. Digital numbers on panel A indicate the number of nodulated plants per total number of GFP-positive plants. **(B)** Numbers of nodules produced per transgenic roots 21 DPI with rhizobia. **(C)** Nitrogenase activities of transgenic nodules 21 DPI with rhizobia determined using acetylene reduction method. ^**^P < 0.01 (*t*-test). **(D)** Frequency of nodules and bumps generated on the transgenic roots.

To analyze the symbiotic function of the ED of SYMRK, the ED of LjSYMRK was replaced with the ED of MtDMI2, OsSYMRK, and AtSYMRK to obtain the chimeric protein MtDMI2^ED^-LjSYMRK^CD^, OsSYMRK^ED^-LjSYMRK^CD^, and AtSYMRK^ED^-LjSYMRK^CD^, respectively (Figure S3). After transgenically expressed in *symrk-409* roots, the function of these three chimeric proteins in nodulation were analyzed. All these three chimeric proteins could confer significant number of nodules formed on *symrk-409* roots 21 DPI with rhizobia (Figures 4A,B). However, MtDMI2^ED^-LjSYMRK^CD^-transgenic plants produced 30% bumps with no LacZ activity detected (Figure 4D) and lower nitrogenase activity than the other two transgenic nodules (Figure 4C). The ED of LjSYMRK was also replaced with ED of LjNFR1 or LjNFR5 to generate LjNFR1^ED^-LjSYMRK^CD^ and LjNFR5^ED^-LjSYMRK^CD^, respectively, and transgenically expressed these chimeric proteins in *symrk-409* mutant plants. Surprisingly, normal nodules numbers and significantly high nitrogenase activities were measured in the *symrk-409* mutant transformed with either LjNFR1^ED^-LjSYMRK^CD^ or LjNFR5^ED^-LjSYMRK^CD^ (Figure S4). These data suggest that ED of SYMRK is important but might not be necessary while the CD of LjSYMRK is essential for RNS in *Lotus*.

### MLD and LRR domain have a fine-tune regulation on SYMRK-mediated symbiotic response

**Figure 5:**
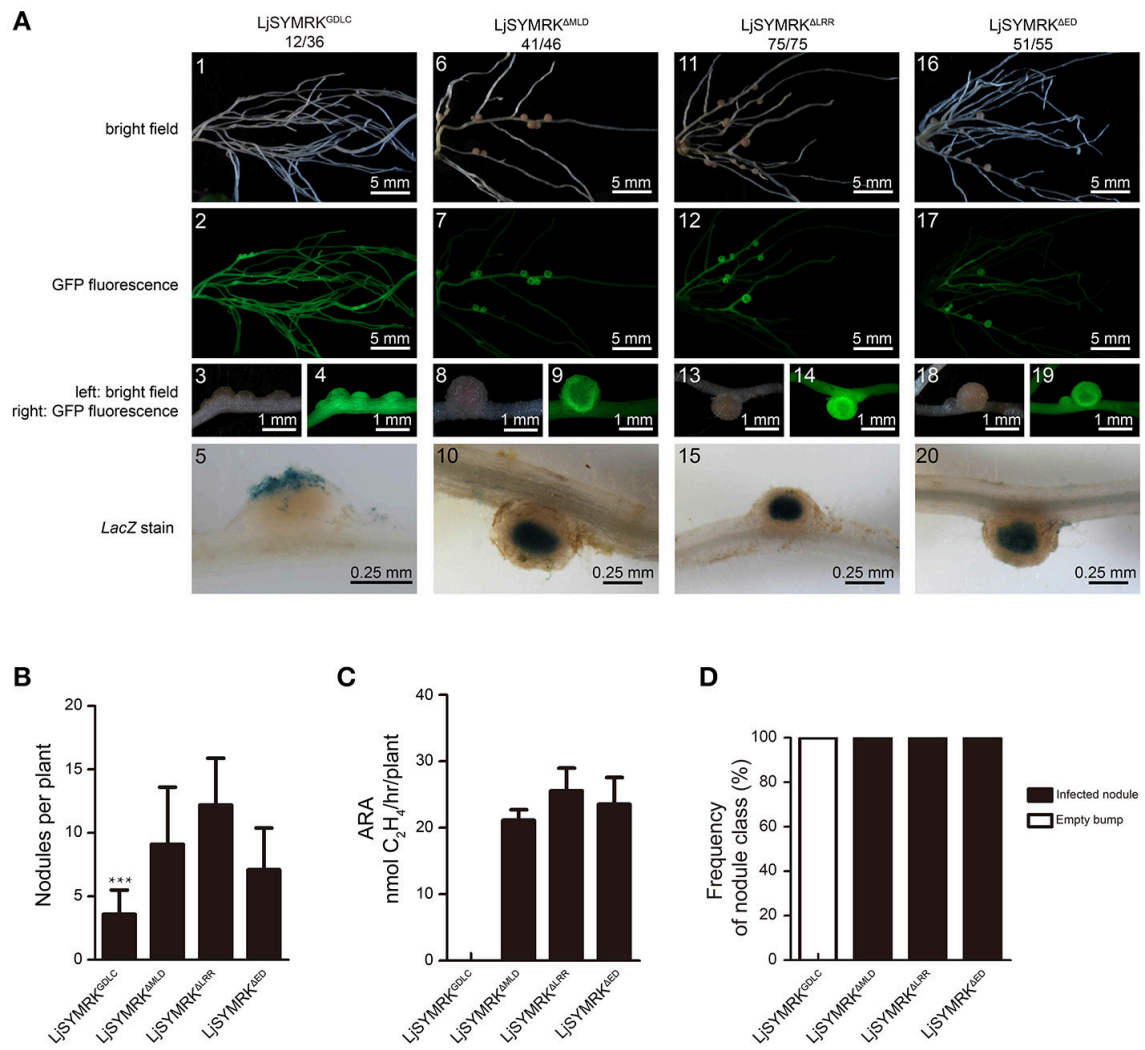
Complementation of *Lotus symrk-409* mutants with LjSYMRK^GDLC^, LjSYMRK^Δ*MLD*^, LjSYMRK^Δ*LRR*^, and LjSYMRK^Δ*ED*^. **(A)** Representative micrographs of different transgenic nodules. Positive transgenic roots identified using GFP signals were inoculated with *M. loti* strain NZP2235 containing a *LacZ* report gene. Nodules and bumps were stained with X-gal. Images showed nodules produced on *symrk-409* roots transgenically expressed LjSYMRK^GDLC^ **(A1–5)**, LjSYMRK^Δ*MLD*^ **(A6–10)**, LjSYMRK^Δ*LRR*^ **(A11–15)**, and LjSYMRK^Δ*ED*^ **(A16–20)**. Digital numbers on panel A indicate the number of nodulated plants per total positive roots. **(B)** Numbers of nodules and bumps produced on the transgenic roots 21 DPI with rhizobia. ^***^P < 0.01 (*t*-test). **(C)** Nitrogenase activities of transgenic nodules 21 DPI with rhizobia determined using acetylene reduction method. **(D)** Frequency of nodules and bumps generated on the transgenic roots.

Complementation with either MtDMI2 or MtDMI2^ED^-LjSYMRK^CD^ in *symrk-409* plants could produce both effective nodules and ineffective bumps. To test which domain is required for its function in negative regulate rhizobia infection, we made four truncated versions of LjSYMRK (Figure S3), i.e., LjSYMRK^Δ*ED*^ (LjSYMRK without ED), LjSYMRK^Δ*MLD*^ (LjSYMRK without MLD), LjSYMRK^Δ*LRR*^ (LjSYMRK without LRR domain and “GDPC” motif), and LjSYMRK^GDLC^ (Proline replaced with leucine). LjSYMRK deleted with MLD, or LRR, or ED could rescue the defects on nodulation phenotype in *symrk-409* mutant plants (Figures 5A5–20). The transgenic plants with overexpression of LjSYMRK^Δ*MLD*^, or LjSYMRK^Δ*LRR*^, or LjSYMRK^Δ*ED*^ formed a significant number of nodules with high nitrogenase activities measured (Figures 5B–D), suggesting that the EDs of SYMRK might not be necessary to be involved in symbiosis. However, LjSYMRK^GDLC^ (a mutation at the conserved residues “GDPC” motif) whose MLD was not detected to be cleaved during symbiosis could not completely complement *symrk-409* mutant plants with only ineffective bumps developed (Figures 5A1–5,B–D). These data suggest that MLD or LRR domain alone is dispensable but might work together to reduce rhizobial infection in mutant plants expressing LjSYMRK^GDLC^.

### The LRR domain plays a crucial role in LjSYMRK^GDLC^-mediated RNS

**Figure 6:**
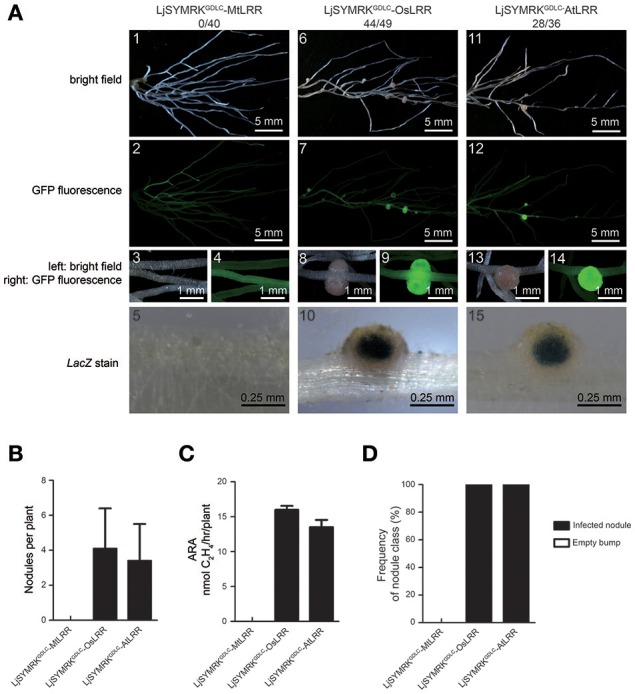
Complementation of *Lotus symrk-409* mutants with LjSYMRK^GDLC^-MtLRR, LjSYMRK^GDLC^-OsLRR, and LjSYMRK^GDLC^-AtLRR. **(A)** Representative micrographs of different transgenic nodules. Positive transgenic roots identified using GFP signals were inoculated with *M. loti* strain NZP2235 containing a *LacZ* report gene. Nodules and bumps were stained with X-gal. Images showed nodules produced on *symrk-409* roots transgenically expressed LjSYMRK^GDLC^-MtLRR **(A1–5)**, LjSYMRK^GDLC^-OsLRR **(A6–10)**, and LjSYMRK^GDLC^-AtLRR **(A11–15)**. Digital numbers on **(A)** indicate the number of nodulated plants per total positive roots. **(B)** Numbers of nodules and bumps produced on the transgenic roots 21 DPI with rhizobia. **(C)** Nitrogenase activities of transgenic nodules 21 DPI with rhizobia determined using acetylene reduction method. **(D)** Frequency of nodules and bumps generated on the transgenic roots.

A previous conclusion indicated that mutation at the GDPC motif that connects MLD and LRR disrupts the cleavage of MLD from SYMRK leading to the block of RNS in *Lotus*. We sought to examine whether LRR domain is required for its function in symbiosis. Chimeric constructs containing LjSYMRK^GDLC^ with LRR domains of MtDMI2, OsSYMRK, and AtSYMRK were made to generate LjSYMRK^GDLC^-MtLRR, LjSYMRK^GDLC^-OsLRR, and LjSYMRK^GDLC^-AtLRR, respectively (Figure S3), and functionally tested in *symrk-409* mutant plants using hairy root transformation approach. Transgenic roots overexpressing either LjSYMRK^GDLC^-OsLRR or LjSYMRK^GDLC^-AtLRR but not LjSYMRK^GDLC^-MtLRR could generate effective nodules with high nitrogenase activity per root tested (Figure 6). These findings implicate that the LRR domain is critical among SYMRK homologous proteins in the RNS.

**Figure 7:**
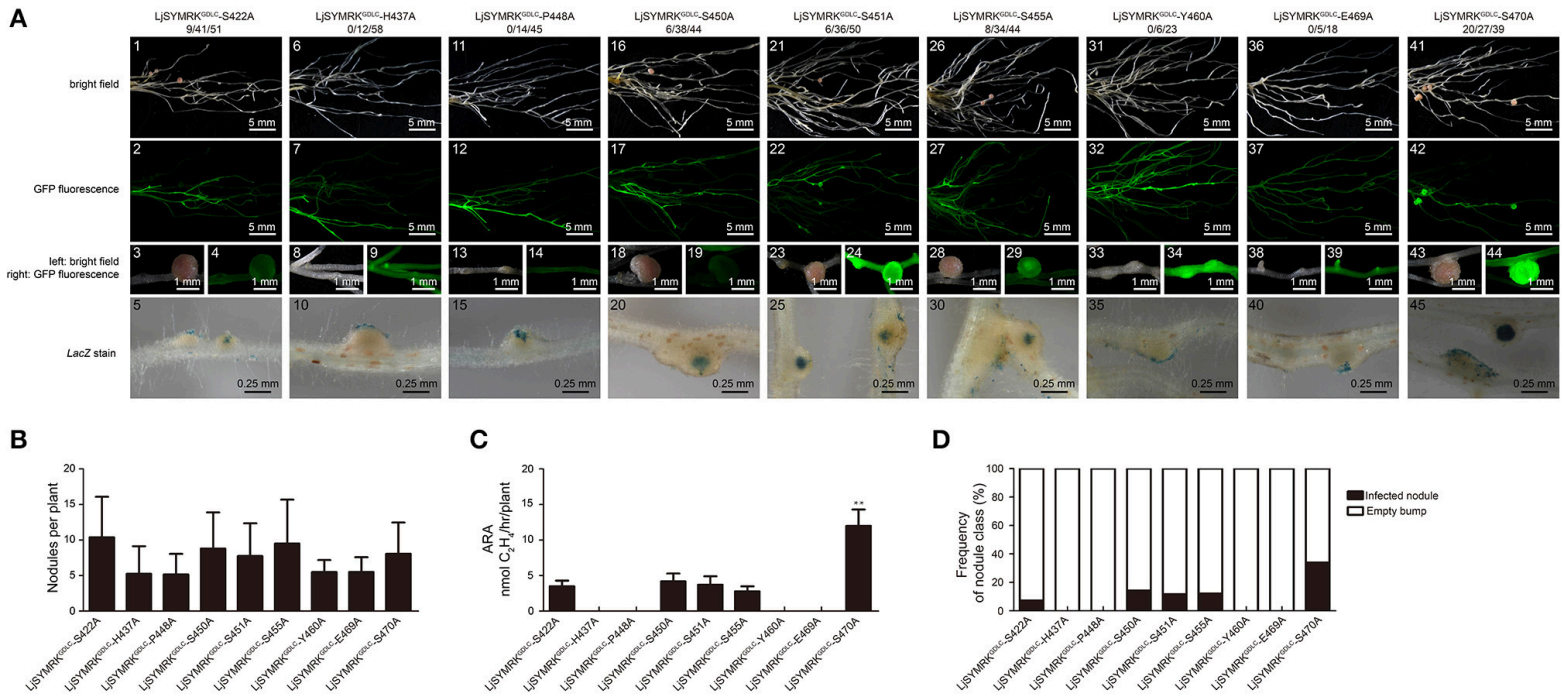
Complementation of *Lotus symrk-409* mutants with constructs of point mutations on the LRR domain of LjSYMRK^GDLC^. **(A)** Representative micrographs of different transgenic nodules. Positive transgenic roots identified using GFP signals were inoculated with *M. loti* strain NZP2235 containing a *LacZ* report gene. Nodules and bumps were stained with X-gal. Images showed nodules produced on *symrk-409* roots transgenically expressed LjSYMRK^GDLC^-S422A **(A1–5)**, LjSYMRK^GDLC^-H437A **(A6–10)**, LjSYMRK^GDLC^-P448A **(A11–15)**, LjSYMRK^GDLC^-S450A **(A16–20)**, LjSYMRK^GDLC^-S451A **(A21–25)**, LjSYMRK^GDLC^-S455A **(A26–30)**, LjSYMRK^GDLC^-Y460A **(A31–35)**, LjSYMRK^GDLC^-E469A **(A36–40)**, and LjSYMRK^GDLC^-S470A **(A41–45)**. Digital numbers on **(A)** indicate the number of nodulated plants per total positive roots. **(B)** Numbers of nodules and bumps produced on the transgenic roots 21 DPI with rhizobia. **(C)** Nitrogenase activities of transgenic nodules 21 DPI with rhizobia determined using acetylene reduction method. ^**^P < 0.01 (*t*-test). **(D)** Frequency of nodules and bumps generated on the transgenic roots.

In order to verify the importance of LRR domains, the alignment of amino acid sequence of LRR domains of SYMRK from different plant species were made (Figure S5) and nine conserved residues were chosen for point mutation into alanine based on the construct containing LjSYMRK^GDLC^. All these variants were transgenically overexpressed in *symrk-409* mutant plants and nodulation phenotype was assayed 21 DPI with rhizobia. Transgenic roots expressing LjSYMRK^GDLC^ with mutations at H437, P448, Y460, and E469, respectively, only developed ineffective nodules with no nitrogenase activity detected (Figure 7), which is similar to the phenotype on LjSYMRK^GDLC^-expressing transgenic roots. However, mutations at five serine residues (S422, S450, S451, S455, and S470) in LjSYMRK^GDLC^ induced the formation of nodules in *symrk-409* mutant plants, some of which are rhizobia-infected nodules with measurable nitrogenase activity (Figure 7), indicating that these five serine residues are critical in SYMRK^GDLC^.

## Discussion

SYMRK protein plays a crucial role in root nodule symbiosis in leguminous plants in response to compatible rhizobia. In this study, domain swap approach was used to study the function of different domains of SYMRK involved in RNS in *Lotus*. The broad goal of this study was to refine the molecular function of different domains of SYMRK in the contribution to root nodule symbiosis using domain swap approach in *Lotus japonicus*.

SYMRK is a key component in the symbiotic signaling pathway, which is essential for both RNS and AMS. In leguminous plants, such as in *Medicago*, MtDMI2 is required for both RNS and AMS, while in rice, OsSYMRK was only shown to be required for AMS and AtSYMRK was shown not to be involved in symbiosis in *Arabidopsis*. To test the roles of different domains of SYMRK in RNS, several chimeric SYMRK protein-encoding genes were constructed under the control of the *Ljubiquitin* promoter and multiple individual transgenic roots were used to test their functions in *Lotus*. The diverse function of SYMRK homologous proteins was further confirmed with the finding that MtDMI2 and OsSYMRK but not AtSYMRK could complement *Lotus SYMRK*^−/−^ mutant to mediate RNS. The function of SYMRK proteins were tested in *Lotus SYMRK*^−/−^ plants in response to rhizobia. The partial complementation by MtDMI2 by forming some ineffective nodules suggests the slight different function of SYMRK between *Lotus* and *Medicago* might exist. Since *Medicago* forms indeterminate nodules while *Lotus* forms determinate nodules with rhizobia, it is possible that SYMRK-mediated RNS have a little difference within these two plant species. Domain swap approach by replacing the CD of LjSYMRK with CDs from other SYMRK homologs confirmed that AtSYMRK is not functional in Lotus to mediate RNS. While the ED of LjSYMRK seems to be not so important since replacing LjSYMRK^ED^ with ED from other SYMRK proteins or even from NFR1 and NFR5 is still functional to mediate RNS in *Lotus*. The critical function of SYMRK^CD^ in symbiosis was also reported that spontaneous nodule formation was observed in *Medicago* and *Lotus* when the kinase domain of AhSYMRK and the full length LjSYMRK were overexpressed, respectively (Ried et al., 2014; Saha et al., 2014). Chimeric protein AtSYMRK^ED^-LjSYMRK^CD^ but not full length of AtSYMRK could complement *Lotus SYMRK1*^−/−^ mutants indicates that the kinase domain of SYMRK is critical to induce nodule initiation even regardless of its ED or without rhizobia treatment when overexpressed in plants.

The ED of SYMRK contains two important domains, i.e., MLD and LRR linked by a GDPC motif. The well-established model about SYMRK reveals that cleavage of MLD is required for its association with NFR5 to mediate symbiotic signaling transduction in *Lotus* (Antolín-Llovera et al., 2014). It seems that the presence of MLD suppresses SYMRK-mediated symbiotic signaling since *Lotus symrk-14* mutants having a point mutation at the linker (“GDPC” motif was mutated into “GDLC”) between MLD and LRR showed strong inhibition on bacterial infection but slight suppression on the development of nodules (Ried et al., 2014). The abnormal function of SYMRK-14 (or SYMRK^GDLC^) in RNS was exaggerated with only ineffective nodules formed in *Lotus* when SYMRK^GDLC^ was overexpressed. However, overexpression of SYMRK^GDLC^ with deletion of either MLD or LRR domain only produced effective nodules, suggesting that both MLD and LRR domain of SYMRK might play a negative rule but might work together in SYMRK^GDLC^-mediated nodule organogenesis in *Lotus*. The same phenotype with effective nodule formation was also observed in Lotus when LjSYMRK^GDLC^ with its LRR replaced by the LRR domains from OsSYMRK or AtSYMRK but not from MtDMI2 was overexpressed, suggesting that LRR domains from OsSYMRK or AtSYMRK might be not functional in mediating RNS in Lotus. The negative role of LRR domain in SYMRK^GDLC^-mediated response is further confirmed by point mutations at five conserved serine residues, leaving us a possibility that the negative function of LRR might be regulated by an unknown phosphorylation.

The ED of SYMRK seems to have a fine-tune regulation on the activation of symbiotic signaling in *Lotus*. Replacement ED with EDs from OsSYMRK or AtSYMRK proteins or even with NFR1 and NFR5 could contribute symbiotic response when overexpressed in *Lotus* plants. Since these proteins located far from LjSYMRK based on the phylogenetic tree, it is possible that these chimeric proteins with nonfunctional EDs still keep some function as LjSYMRK^Δ*ED*^ to induce symbiotic response. However, the chimeric protein MtDMI2^ED^-LjSYMRK^CD^ could not completely complement Lotus *SYMRK*^−/−^ plants, indicating that the functional difference between *Lotus* and *Medicago* possibly due to that different nodule types formed on these two plants. The abovementioned hypothesis is further confirmed by the finding that LjSYMRK^GDLC^-MtLRR but not LjSYMRK^GDLC^-AtLRR or LjSYMRK^GDLC^-OsLRR is not functional to mediate RNS in *Lotus*. The whole ED from LjSYMRK might be nonfunctional when LRR domain was replaced with LRR from AtSYMRK or OsSYMRK. However, since the LRR from MtDMI2 might have difference to mediate RNS, the MLD of LjSYMRK seems not to match with the LRR from MtDMI2 which finally made the chimeric protein LjSYMRK^GDLC^-MtLRR lose its function in RNS. The data indicated that MLD and LRR must have a fine-tune regulation on the activation of symbiotic response.

In conclusion, the intracellular domain of SYMRK is critical while the ED of SYMRK plays a relatively minor role in mediating RNS in *Lotus*. GDPC motif that connects MLD and LRR domain required for MLD cleavage in response to RNS is responsible for nodule organogenesis. Both MLD and LRR domains might have a fine-tune regulation of SYMRK-mediated nodule organogenesis in *Lotus*. This progress in defining the molecular functions of SYMRK in RNS. However, identification of the direct targets of SYMRK and crystal structure of SYMRK are the future topics for understanding the molecular mechanisms mediated by SYMRK in NRS.

## Materials and methods

### Homology analysis

SYMRK homologous proteins were identified based on the sequence similarity to *Lotus* SYMRK with the protein blast suite at the National Center for Biotechnology Information database (https://blast.ncbi.nlm.nih.gov) used. The phylogenetic tree was built by MEGA 5.1 software (Tamura et al., 2011).

### Plant growth and rhizobia inoculation

*Lotus* seeds (B-129 Gifu and *symrk* mutant) and hair root transgenic seedlings were grown in vermiculite and perlite mixed (2:1 volume ratio) supplied a 1/2 B&D medium and placed in a growth chamber set at 22°C for 16-h-light/8-h-dark cycle. Plants were inoculated with *Mesorhizobium loti* strain NZP2235 expressing beta-galactosidase (LacZ) after 5 days. Rhizobia grown in liquid TY medium adding tetracycline at OD_600_ of 1.0. They were then pelleted and resuspended in 1/2 B&D medium containing 0.5 mM KNO_3_ at OD_600_ of 0.02.

*Medicago* hair root transgenic seedlings were grown in vermiculite and perlite mixed (2:1 volume ratio) supplied a 1/2 FM medium and placed in a growth chamber set at 22°C for 16-h-light/8-h-dark cycle. Plants were inoculated with *Sinorhizobium meliloti* strain Sm2011 expressing LacZ after 5 days. Rhizobia grown in liquid TY medium adding tetracycline at OD_600_ of 1.0. They were then pelleted and resuspended in 1/2 FM medium containing 0.5 mM KNO_3_ at OD_600_ of 0.02.

### *Lotus symrk* mutant identification

Genomic DNA was extracted from putative mutant plants and used for PCR amplification of 2 min soak at 95°C, followed by 35 cycles of 30 s at 94°C, 30 s at 58°C, and 50 s at 72°C, followed by 5 min soak at 72°C. Primers for genotyping of *symrk-409* insertion alleles are provided in Table S1, which were performed as described previously (Fukai et al., 2012; Urbanski et al., 2012).

### RNA isolation and quantitative RT-PCR

Total RNA was extracted from roots at 7 DPI using the EASYspin Plant RNA Kit (Aidlab, China). Primescript RT Reagent Kit (TaKaRa, Japan) was used to remove gDNA of RNA samples and reverse transcribe RNA into cDNA. Quantitative real-time PCR was performed on an ABI ViiA^TM^7 Real-Time PCR System (ABI, USA) using One-Step SYBR PrimeScript RT-PCR kit II (Takara, Japan). *Lotus ATP synthase* (Genbank ID: AW719841) and *ubiquitin* (Genbank ID: AW720576) were used as reference genes, which are stably expressed in all plant tissues. Primers for qRT-PCR are listed in Table S1.

### Plasmid construction for mutant complementation

The pUB-GFP vector was digested by restriction endonucleases *Xba*I and *Stu*I, then linked to a fragment or multi-fragments by Gibson assembly (Gibson et al., 2009). Primers for PCR are listed in Table S1.

### *Lotus* hairy root transformation

The pUB-GFP vectors carrying a variety of chimeric SYMRK constructs were transformed into *symrk-409* seedlings using *Agrobacterium rhizogenes* LBA1334 as described by Wang et al. (2013). The GFP marker was used for selection of transgenic hairy roots by a fluorescence stereo microscope (Nikon SMZ18, Japan).

### *Medicago* hairy root transformation

Transgenic root on *dmi2-1* seedlings were induced using *A. rhizogenes* MSU440 as described by Wang et al. (2016). Positive transgenic hairy roots were identified by GFP marker using a fluorescence stereo microscope (Nikon SMZ18, Japan).

### X-Gal staining

Plants were inoculated with rhizobia carrying a *lacZ* reporter gene. At 7 or 21 DPI, roots were vacuum infiltrated for 5 min in fixative solution (1.25% Glutaraldehyde dissolved in 0.1 M potassium phosphate buffer pH 7.4), and placed at room temperature for 40 min, washed twice 10 min in 0.1 M potassium phosphate buffer. Roots were vacuum infiltrated for 5 min with X-Gal staining solution [0.1 M phosphate buffer, 6.25 mM K_4_Fe(CN)_6_, 6.25 mM K_3_Fe(CN)_6_, 0.75% X-Gal in DMF] and kept overnight at 28°C in the dark. Roots were washed twice in 0.1 M potassium phosphate buffer for 5 min, then rinsed twice in ddH_2_O for 5 min. Stained roots were examined using a fluorescence stereo microscope (Nikon SMZ18, Japan) and a fluorescence microscope (LEICA DM2500, Germany).

### Detection of nitrogenase activity

Acetylene reduction activity (ARA) was used to detect nitrogenase activity of root nodules at 21 DPI. Four nodulated seedlings were transferred into test tubes with 2 ml acetylene added for additional growth at 28°C for 2 h. At least three repeats of each experiment were analyzed. Acetylene was surveyed using a GC-4000A gas chromatograph (Dongxi Company, China).

## Accession numbers

Sequence data from this work can be found under the following GenBank accession numbers: AAV88623.1 for SrSYMRK, XP_004512550.1 for CaSYMRK, CAD22013.1 for MaSYMRK, CAD10812.1 for PsSYMRK, XP_003517193.1 for GmSYMRK, CAD10811.1 for MtSYMRK, AAM67418.1 for LjSYMRK, NP_001234869.1 for SlSYMRK, NP_001105860.1 for ZmSYMRK, XP_015646949.1 for OsSYMRK, XP_014752741.1 for BdSYMRK, XP_002460874.1 for SbSYMRK, XP_020677592.1 for DcSYMRK, NP_564904.1 for AtSYMRK, XP_020890981.1 for AlSYMRK, XP_010511724.1 for CsSYMRK, XP_009105317.1 for BrSYMRK, and XP_018445268.1 for RsSYMRK.

## Author contributions

HL, YC, and ZZ designed the experiments and analyzed the results. HL, MC, TZ, and LD performed the experiments and analyzed the data. HL, YC, and ZZ wrote the paper.

### Conflict of interest statement

The authors declare that the research was conducted in the absence of any commercial or financial relationships that could be construed as a potential conflict of interest.
