# Supplementary material for: Domain Swap Approach Reveals the Critical Roles of Different Domains of SYMRK in Root Nodule Symbiosis in Lotus japonicus
Source: Front Plant Sci. 2018 Jun 5;9:697. doi: 10.3389/fpls.2018.00697 (PMC6024595; doi:10.3389/fpls.2018.00697)
Supplement: Supplementary file 2 [file Presentation_1.pdf]

SUPPLEMENTARY MATERIAL

Figure S1. Identification and phenotype of the *L. japonicus symrk-409* mutant.

(A) The exons are shown as black boxes in the schematic diagram of *SYMRK* gene. The insertion site of the *LORE1* insertion mutant *symrk-409* (30010361) is shown. (B) Determine of the wild type (WT), heterozygosity and homozygosity of mutant lines. Primers F/R are designed to amplify the un-insertion fragment, and F/P2 to amplify the inserted genome. The PCR products are the same sizes as expected fragments and also confirmed by DNA sequencing. (C) The expression level of *SYMRK* are quantified by qRT-PCR in 7 dpi root of WT and *symrk-409* mutant. (D-G) Gifu-WT and *symrk-409* inoculated with *M. loti* strain NZP2235, which constitutively expresses beta-galactosidase (*LacZ*). The infection threads are formed on Gifu-WT root (D) and not observed on *symrk-409* root (E) at 7 days after inoculation with rhizobia. The nodules are observed on Gifu-WT root (F) and absence on *symrk-409* root (G) at 21 days after inoculation with rhizobia.

Fig S2. Complementation of *Mtdmi2-1* mutants with LjSYMRK and MtDMI2.

(A) Representative micrographs of different transgenic nodules. Positive transgenic roots identified using GFP signals were inoculated with *S. meliloti* strain Sm2011 containing a *LacZ* report gene. Nodules and bumps were stained with X-gal. Images showed nodules produced on *dmi2* roots transgenically expressed with control vector (A1-A5), MtDMI2 (A6-A10), and LjSYMRK (A11-A15). Digital numbers on top of panel A indicate the number of nodulated plants out of the positive transgens.
(B) Numbers of nodules and bumps generated on different transgenic roots 21 DPI with rhizobia.

(C) Nitrogenase activity of root nodules 21 DPI with rhizobia determined using

acetylene reduction method.

(D) Frequency of nodules and bumps produced on the transgenic roots.

Figure S3. Schematic representation of different SYMRK proteins and chimeric domain SYMRK proteins used for transformation of *L. japonicus* SYMRK mutant.

All constructs were expressed in mutants under the control of *Ljubiquitin* promoter. Different species of SYMRK and domains were indication in different colour: *L. japonicus,* black; *M. truncatula,* blue; *O. sativa,* green; *A. thaliana,* red.

Fig S4. Complementation of *Lotus symrk-409* mutants with LjNFR1^{ED}-LjSYMRK^{CD}, and LjNFR5^{ED}-LjSYMRK^{CD}.

(A) Representative micrographs of different transgenic nodules. Positive transgenic roots identified using GFP signals were inoculated with *M. loti* strain NZP2235 containing a *LacZ* report gene. Nodules and bumps were stained with X-gal. Images showed nodules produced on *symrk-409* roots transgenically expressed with LjNFR1^{ED}-LjSYMRK^{CD} (A1-A5), and LjNFR5^{ED}-LjSYMRK^{CD} (A6-A10). Digital numbers on top of panel A indicate the number of nodulated plants out of the positive transgens.

(B) Numbers of nodules and bumps generated on different transgenic roots 21 DPI with rhizobia.

(C) Nitrogenase activity of root nodules 21 DPI with rhizobia determined using acetylene reduction method.

(D) Frequency of nodules and bumps produced on the transgenic roots.

Fig S5. Sequence alignment of LRR domains among different species

Protein alignment of LRR domain of LjSYMRK and homologous proteins from different legumes and non-legumes. Nine conserved amino acid sites, marked with asterisks, are mutated to alanine in $LjSYMRK^{GDLC}$.

Table S1. List of primers used in this study.
