# Supplementary figures and images for: Domain Swap Approach Reveals the Critical Roles of Different Domains of SYMRK in Root Nodule Symbiosis in Lotus japonicus
Source: Front Plant Sci. 2018 Jun 5;9:697. doi: 10.3389/fpls.2018.00697 (PMC6024595; doi:10.3389/fpls.2018.00697)

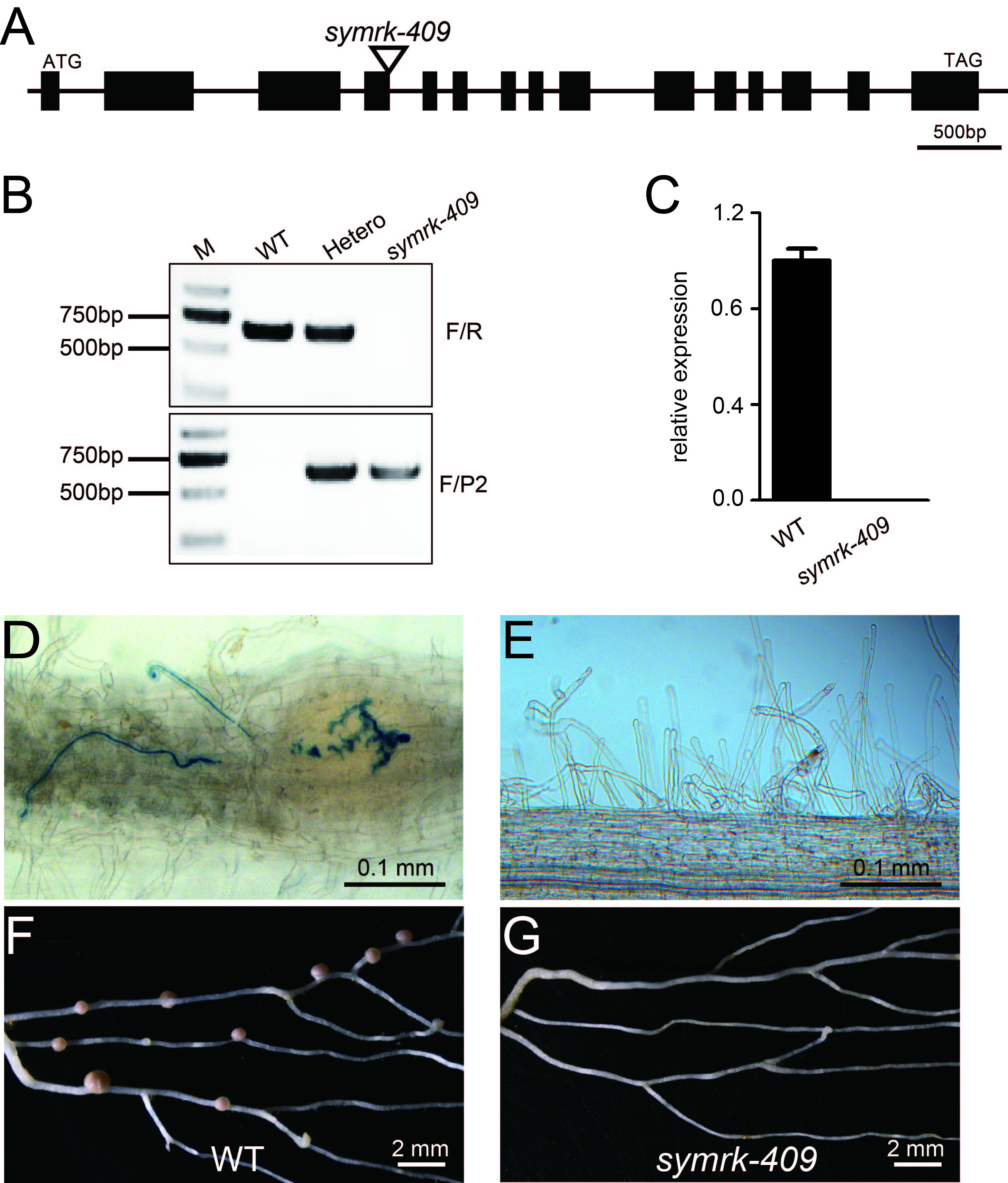

Supplement: Supplementary file 3 [file Presentation_1.zip › figure S1-S5/Fig.S1.jpg]

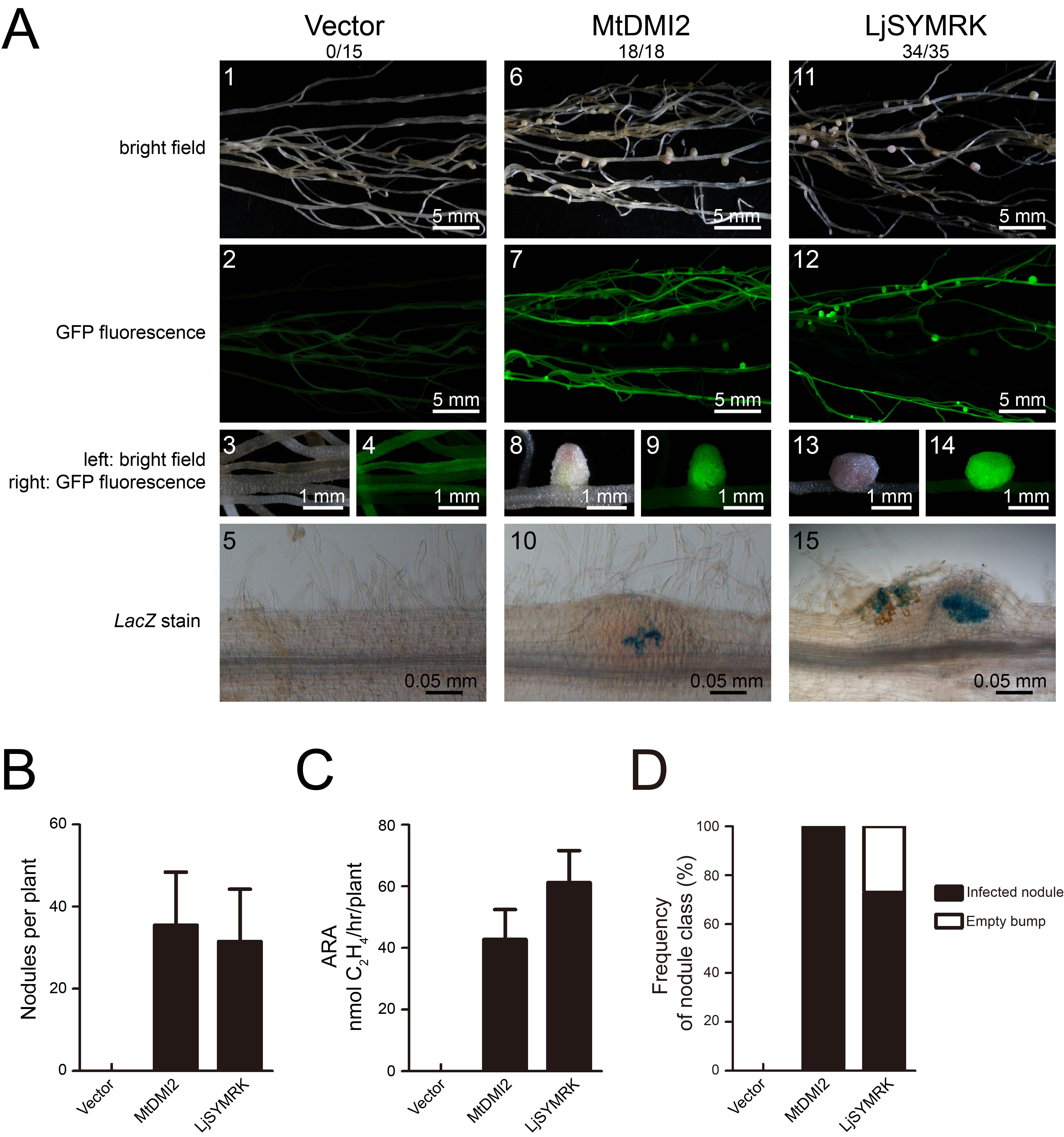

Supplement: Supplementary file 3 [file Presentation_1.zip › figure S1-S5/Fig.S2.jpg]

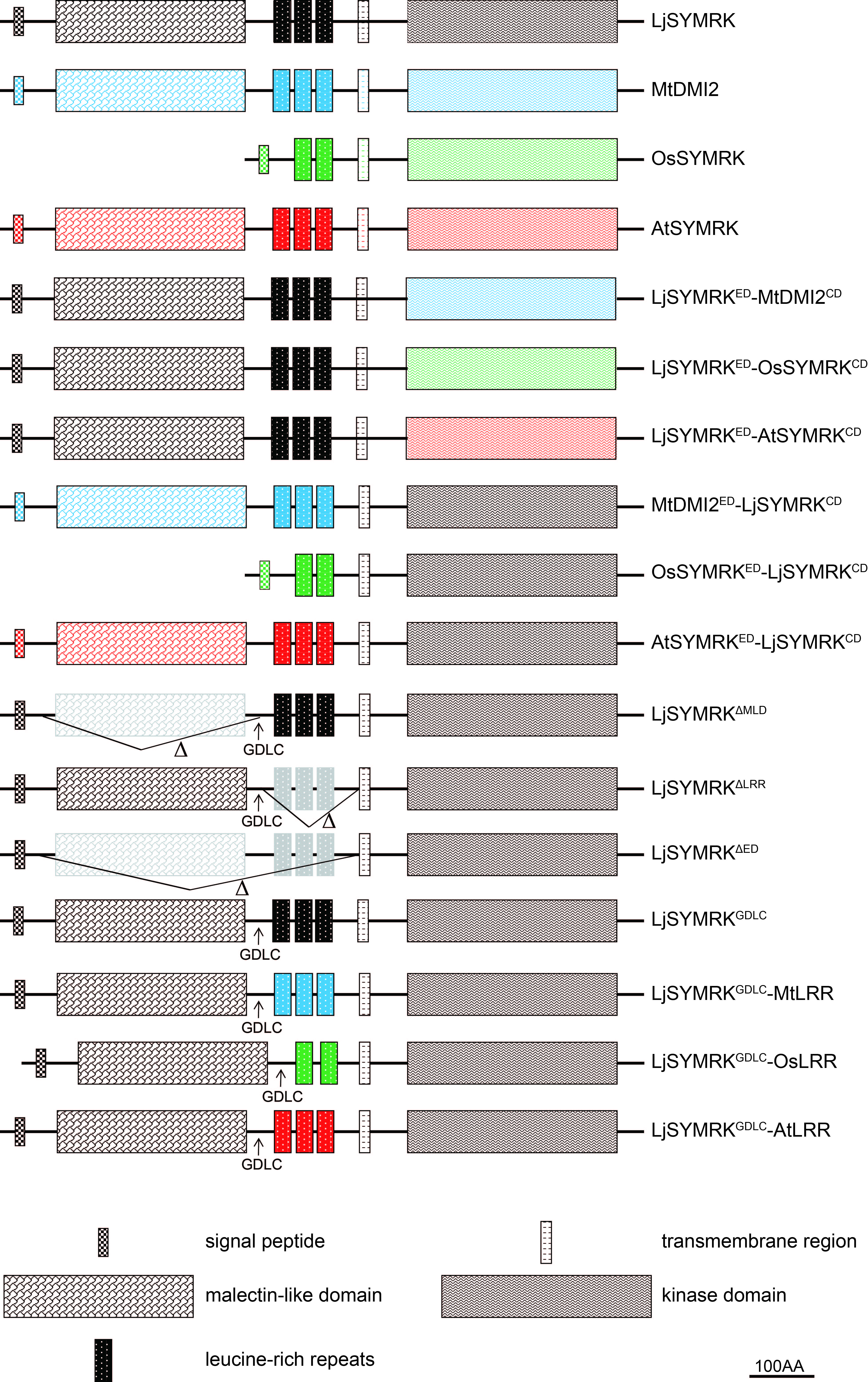

Supplement: Supplementary file 3 [file Presentation_1.zip › figure S1-S5/Fig.S3.jpg]

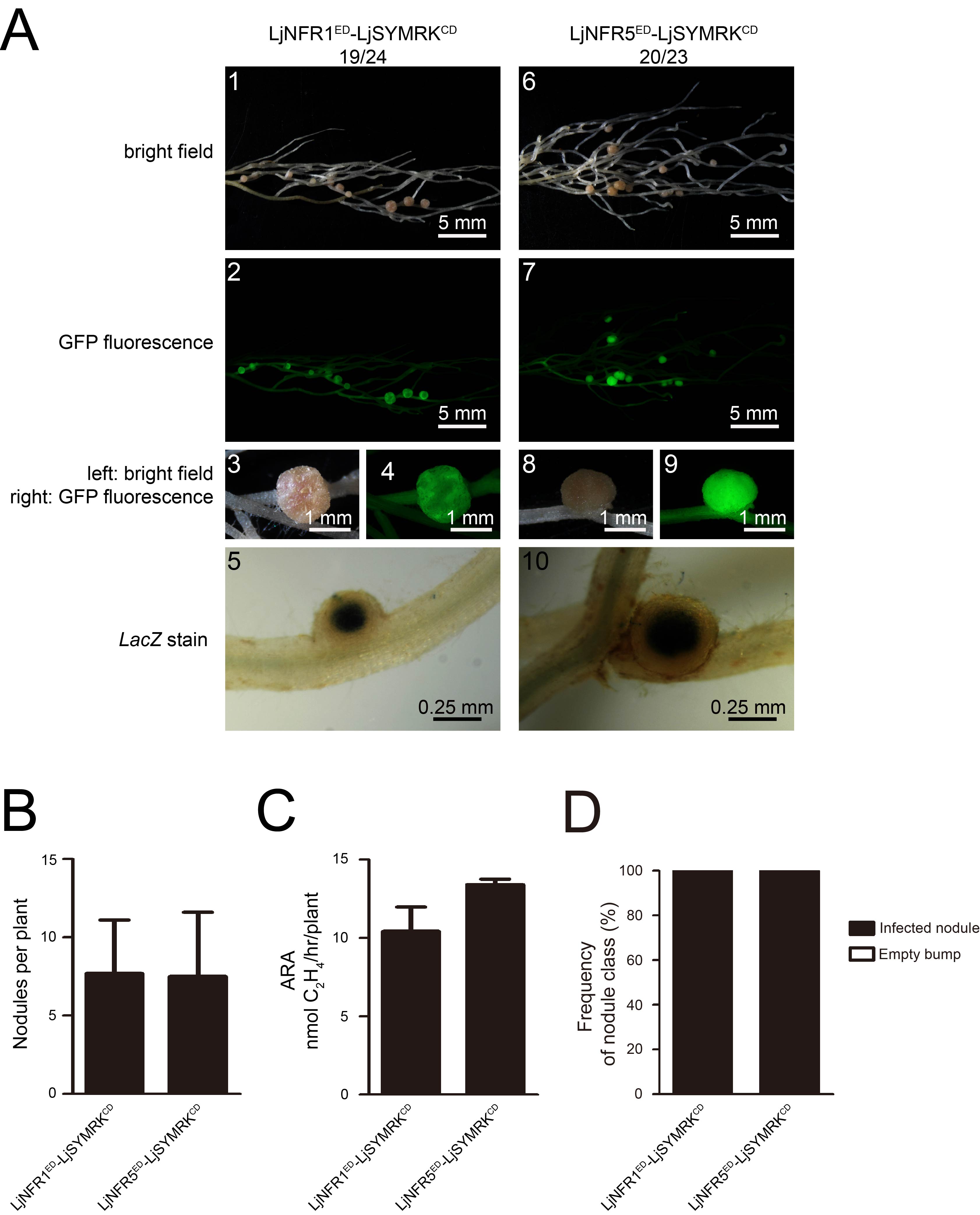

Supplement: Supplementary file 3 [file Presentation_1.zip › figure S1-S5/Fig.S4.jpg]

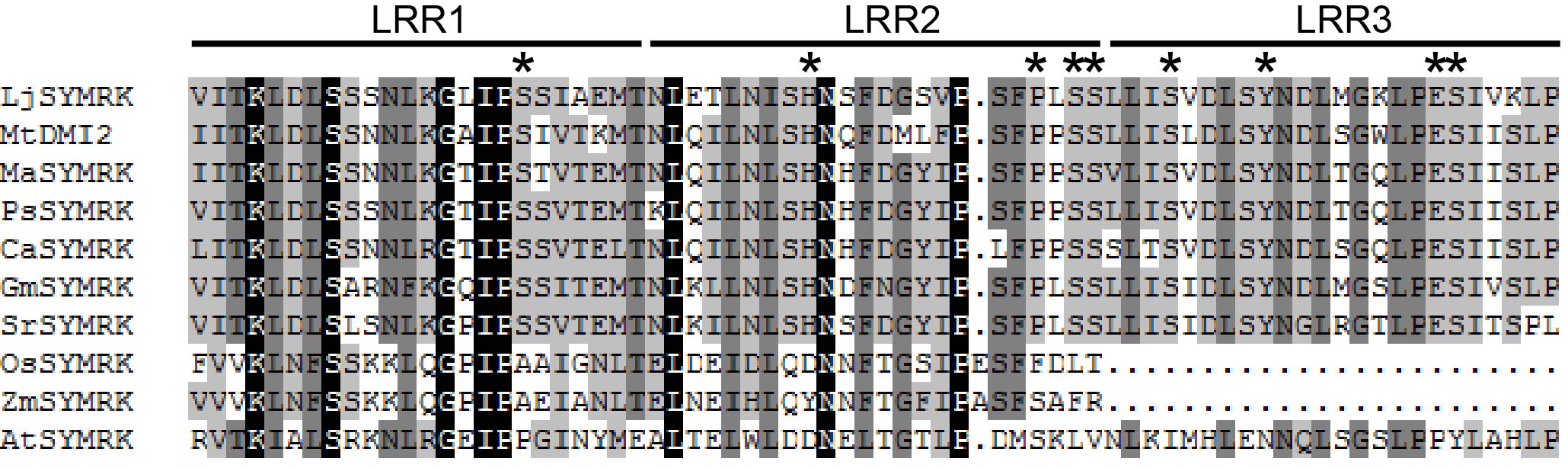

Supplement: Supplementary file 3 [file Presentation_1.zip › figure S1-S5/Fig.S5.jpg]
